# Genome-wide Association Study Identifies New Loci for Resistance to *Leptosphaeria maculans* in Canola

**DOI:** 10.3389/fpls.2016.01513

**Published:** 2016-10-24

**Authors:** Harsh Raman, Rosy Raman, Neil Coombes, Jie Song, Simon Diffey, Andrzej Kilian, Kurt Lindbeck, Denise M. Barbulescu, Jacqueline Batley, David Edwards, Phil A. Salisbury, Steve Marcroft

**Affiliations:** ^1^Graham Centre for Agricultural Innovation (an alliance between NSW Department of Primary Industries and Charles Sturt University), Wagga Wagga Agricultural Institute, Wagga WaggaNSW, Australia; ^2^Diversity Array Technology P/L, University of Canberra, CanberraACT, Australia; ^3^Centre for Bioinformatics and Biometrics, University of Wollongong, WollongongNSW, Australia; ^4^Department of Economic Development, Jobs, Transport and Resources, HorshamVIC, Australia; ^5^School of Plant Biology, University of Western Australia, CrawleyWA, Australia; ^6^Institute of Agriculture, University of Western Australia, CrawleyWA, Australia; ^7^Faculty of Veterinary and Agricultural Sciences, The University of Melbourne, ParkvilleVIC, Australia; ^8^Marcroft Grains Pathology, HorshamVIC, Australia

**Keywords:** natural variation, resistance to *L. maculans*, canola, genome-wide association analysis, linkage analysis, comparative mapping, blackleg, race-specific and race non-specific resistance

## Abstract

Key message “We identified both quantitative and quantitative resistance loci to *Leptosphaeria maculans*, a fungal pathogen, causing blackleg disease in canola. Several genome-wide significant associations were detected at known and new loci for blackleg resistance. We further validated statistically significant associations in four genetic mapping populations, demonstrating that GWAS marker loci are indeed associated with resistance to *L. maculans.* One of the novel loci identified for the first time, *Rlm12*, conveys adult plant resistance in canola.” Blackleg, caused by *Leptosphaeria maculans*, is a significant disease which affects the sustainable production of canola (*Brassica napus*). This study reports a genome-wide association study based on 18,804 polymorphic SNPs to identify loci associated with qualitative and quantitative resistance to *L. maculans*. Genomic regions delimited with 694 significant SNP markers, that are associated with resistance evaluated using 12 single spore isolates and pathotypes from four canola stubble were identified. Several significant associations were detected at known disease resistance loci including in the vicinity of recently cloned *Rlm2*/*LepR3* genes, and at new loci on chromosomes A01/C01, A02/C02, A03/C03, A05/C05, A06, A08, and A09. In addition, we validated statistically significant associations on A01, A07, and A10 in four genetic mapping populations, demonstrating that GWAS marker loci are indeed associated with resistance to *L. maculans*. One of the novel loci identified for the first time, *Rlm12*, conveys adult plant resistance and mapped within 13.2 kb from *Arabidopsis R* gene of TIR-NBS class. We showed that resistance loci are located in the vicinity of *R* genes of *Arabidopsis thaliana* and *Brassica napus* on the sequenced genome of *B. napus* cv. Darmor-*bzh*. Significantly associated SNP markers provide a valuable tool to enrich germplasm for favorable alleles in order to improve the level of resistance to *L. maculans* in canola.

## Introduction

Blackleg disease, caused by the hemibiotrophic fungal pathogen *Leptosphaeria maculans* (Desm.) Ces. et de Not. is a major threat to the consistent global supply of canola (*Brassica napus* L). Canola is sown as a spring crop in the North Americas, winter crop in Europe and autumn crop in Australasia, and contributes approximately 14% of world vegetable oil production (USDA, 2016). Blackleg affects canola plants at various stages of plant development (GS10 to GS80), from cotyledon emergence to pod-filling growth stages. Under severe epiphytotic conditions, *L. maculans* can completely kill the young susceptible seedlings and cause catastrophic yield loss (Marcroft et al., 2002; Fitt et al., 2006).

Two types of resistance to *L. maculans;* qualitative and quantitative, have been reported in *B. napus* (Delourme et al., 2006). Qualitative resistance mediated by an effector–triggered immunity (ETI) mechanism relies on specific interaction between a race-specific *R* protein and corresponding avirulent (*Avr*) protein; also known as the gene-for-gene interaction (Flor, 1971). *B. napus* resistant genotypes do not kill this apoplastic pathogen but limit its biomass and asexual and sexual sporulation (Huang et al., 2006a; Stotz et al., 2014). Eighteen qualitative (*R*) genes for ETI have been identified in *Brassica* species; *B. rapa, B. juncea, B. napus*, and *B. nigra* and several of them are deployed in commercial canola varieties (Delourme et al., 2011; Marcroft et al., 2012; Balesdent et al., 2013; Larkan et al., 2013, 2015; Raman et al., 2013b). Intensive cultivation of broad-acre varieties carrying specific *R* genes results in the emergence of new virulent (*avr)* races and rapid change in the frequency of existing avirulence (*Avr*) alleles in natural populations of *L. maculans*. As a consequence, some *R* genes have become ineffective just within 5 years of their commercial release (Brun et al., 2000; Howlett et al., 2001; Li et al., 2003; Rouxel et al., 2003; Sprague et al., 2006; Van de Wouw et al., 2014).

Quantitative resistance (race non-specific) mediated by pattern–triggered immunity (PTI) mechanism via pathogen associated molecular patterns (Young, 1996; Dangl and Jones, 2001) does not effectively control blackleg disease at the seedling stage, but restrict the development of canker formation in mature stems (Delourme et al., 2006). However, knowledge of loci, including their uniqueness in controlling quantitative resistance under different environment conditions is rather limited in *B. napus* germplasm (Delourme et al., 2006; Huang et al., 2009; Kaur et al., 2009; Jestin et al., 2011; Raman et al., 2012). Australia has the most diverse population of *L. maculans* compared to other canola growing countries (Hayden et al., 2007) but host–pathogen interaction under the Australian environment is poorly understood due to G × E interactions and highly heterogeneous populations of *L. maculans* across canola growing regions. Understanding the genetic bases of resistance to *L. maculans* among diverse canola varieties will allow canola breeders to produce elite varieties with durable resistance, and design blackleg management strategies to achieve a high yield to meet the demand of a growing human population.

Both linkage and genome-wide association mapping (Flint-Garcia et al., 2003; Jannink, 2007) has been used simultaneously to unravel loci and candidate genes associated with resistance to *L. maculans* and for other traits of agricultural significance in canola (Raman et al., 2013a; Cai et al., 2014; Fopa Fomeju et al., 2014; Li et al., 2014; Raman H. et al., 2016; Raman R. et al., 2016; Wang et al., 2016). Single spore isolates (SSI) have been extensively utilized to map and characterize loci involved in resistance to *L. maculans*. However, the majority of individual isolates representing the Australian differential set have multiple *Avr* genes (Marcroft et al., 2012). These isolates could be used to identify and validate molecular marker loci for resistance in diverse canola accessions, e.g., GWAS panels, rather than following extremely laborious, low-resolution mapping of bi-parental populations. Several disease resistance genes have been identified in canola and in *Arabidopsis* – *L. maculans* pathosystem (Staal et al., 2006, 2008; Staal and Dixelius, 2008; Persson et al., 2009; Larkan et al., 2013, 2015) and would provide insights on the genetic architecture of qualitative and quantitative resistance loci in canola.

We determine the extent of genetic variation in resistance to *L. maculans* utilizing a panel of 179 diverse accessions of canola (Raman H. et al., 2016) and reveal genetic loci for race-specific and race non-specific resistance using a GWA strategy. We identified at least 503 markers associated with qualitative and quantitative resistance to *L. maculans* under the glasshouse and greenhouse experiments. We then validated the genome-wide SNP associations using linkage analyses of four doubled haploid (DH) populations derived from AG-Castle^∗^3/Westar-10 (*Rlm3*), BLN2762/Surpass400 (*Rlm4, LepR3*), Maxol^∗^3/Westar-10 (*Rlm1* and *Rlm3*) and Skipton/Ag-Spectrum (*Rlm4* and *Rlm12*). We further locate the physical map positions of the significant genomic regions on the *B. napus* sequenced genome, associated with resistance in relation to the candidate genes implicated in effector-triggered immunity in *Arabidopsis* and *B. napus*. Our work demonstrates the usefulness of GWAS in the discovery of new putative resistance loci to *L. maculans*, including reporting the *Rlm12* locus for the first time.

## Materials and Methods

### Evaluation of Diverse Accessions and DH Lines with SSI

A diverse GWAS panel of 179 accessions of *B. napus* (Raman H. et al., 2016) was evaluated for resistance to 12 SSI (04MGPS021, 06MGPP041, D8, D9, IBCN13, IBCN15, IBCN16, IBCN17, IBCN18, IBCN75, IBCN76, and PHW1223) of *L. maculans* (Marcroft et al., 2012; Raman R. et al., 2013) at the Wagga Wagga Agricultural Institute. Details of *Avr* gene profiles of the SSI were described previously (Marcroft et al., 2012; Zander et al., 2013). Plants were grown in a randomized block designs generated using the software package DiGGer (Coombes, 2002) to randomize genotypes to tray blocks and spatially arrange genotypes within trays. For glasshouse experiments, seedlings were raised in plastic trays (7 × 8 cells) and inoculated as described previously in Raman et al. (2012).

Two replicates of 188 selfed diverse lines were arranged in a 4 row by 94 column array in the glasshouse. Two benches, each holding a replicate, had 4 rows of 47 trays, with each tray holding a row of 8 entries, and each experimental unit in the tray comprising a column of 7 seedlings. After 17–20 days from inoculation, cotyledons were scored on the basis of lesion size for the resistance or susceptibility using a scale between 0 (for highly resistance) to 9 (highly susceptible) (Raman et al., 2012).

In order to assess whether genetic variation for resistance expressed at the cotyledon stage is still effective at the adult plant stage (physiological maturity), five random plants (after the initial cotyledon scores) from each of the 12 SSI evaluations were transplanted in white plastic pots (10 cm diameter) according to a statistical design under greenhouse conditions and were raised till maturity. All plants were cut with secateurs at the crown and assessed for internal infection on a 0 to 100 scale based on per cent area showing necrosis (Raman et al., 2012). We also verified the accuracy of the visual assessment of crown canker lesions after infection with isolate 04MGPS021 which has been extensively used in previous studies (Raman et al., 2012; Zander et al., 2013; Raman et al., in review) by comparing visual scoring with ‘digitized’ internal infection score. The crowns of cut plants were first scored using the blackleg rating scale (0 to 100), and then photographed with a digital camera (Canon EOS 450D). Acquired digital images were measured for discolouration (black/brown areas) using the software^1^ ‘Image J’.

### Evaluation of Diverse Lines with Pathotypes from Stubbles

In order to mimic field evaluation of germplasm and to reduce genotype × environment interaction, we tested the GWAS diversity panel and a backcross DH population from SAgSDH (Raman R. et al., 2016) with pathotypes from stubble using the ‘ascospore shower’ test (Huang et al., 2006b) under glasshouse conditions. Canola stubble from commercial crops of AV-Garnet (MT Hope, South Australia), CB-Jardee HT (Frances, South Australia), Monola76TT (Bool Lagoon, SA, Australia), and ATR-Cobbler (Wagga, NSW, Australia) grown in the 2011 cropping season was used. Sexual spores from pseudothecia were released from each stubble source and sprayed individually with distilled water. Plants inoculated with four different stubble sources were maintained at 100% relative humidity in growth room maintained at 18°C for 96 h and then transferred to greenhouse conditions. A GWAS diversity set was grown using a randomized complete block design with two replications. Each accession was grown in pots with the consistent plant density (4). Inoculated plants were assessed for per cent internal infection at physiological maturity.

The predicted means of disease scores for each genotype were used to determine the extent of genetic variation for resistance to *L. maculans* and to detect genome-wide trait-marker associations. Broad sense heritability was estimated for DH lines in each experiment (isolate/ascopsore shower test) using a method described previously (Cullis et al., 2006).

### Genome-wide Association Analysis

A set of 18,804 single nucleotide polymorphism (SNP) and presence-absence markers with allele frequency >0.05 and call rate >80% was used for identification of trait-marker associations using the Emma/P3D method (Kang, 2008; Zhang et al., 2010; Raman H. et al., 2016) implemented in the R package Genome Association and Prediction Integrated Tool (Lipka et al., 2012). We used principal components (Price, 2006) and relative kinship coefficients (VanRaden, 2008) to reduce spurious associations between trait and markers as described in Raman H. et al. (2016). Genome-wide associations between markers and resistance to *L. maculans* was initially tested at *p* < 0.001. In order to reduce the chance of false positives, we used a Bonferroni correction based on a Type I error rate of 0.05. A stringent Bonferroni correction was calculated as described previously (Li et al., 2014) by dividing 0.05 by the total number of markers 18804 used for the GWAS analysis. Highly significant association between DArTseq markers and resistance to *L. maculans* was ‘declared’ when *p* < 2.66 × 10^-6^ or -log10(*p*) > 5.57. The -log10(*p*) values for each SNP were exported to generate a Manhattan plot.

### Validation of Race-specific and Race Non-specific Resistance Loci

The associations detected through GWAS, were compared against the quantitative trait locus (QTL) marker intervals associated with resistance to *L. maculans* in DH mapping populations (94 to 186 lines) derived from (SASDH (Raman et al., 2012), BSDH (Raman R. et al., unpublished), and Maxol^∗^1/Westar-10 MWDH (Raman R. et al., 2013). Conventional microarray DArT, and SSR markers, which showed linkage with the *Rlm1* locus for resistance to *L. maculans* in a DH population from MWDH (Raman R. et al., 2013) were further integrated with DArTseq markers mapped on 1518 loci (this study). In addition, we mapped two DH populations derived from Ag-Castle^∗^3/Westar-10 (AWDH) and Skipton/Ag-Spectrum//Skipton (SAgSDH).

(i) Mapping of the *Rlm12* Locus in the SAgS Population.

The SAgSDH population comprising 146 lines was evaluated for resistance with the ‘ascospore shower’ test as described above. Mixed stubble was sourced from the current Australian canola cultivars: CrusherTT, CB-Telfer, ATR-Stingray, Hyola50, *Brassica juncea*, and ThumperTT. An incomplete block design, where trays on benches comprise incomplete blocks, was used. Three replicates of 146 DH lines and 42 entries of two parents were arranged in a 20 row by 24 column array in the glasshouse. Seeds were sown in plastic pots accommodating four plants/DH line.

The linkage map of SAgS DH population comprising 7716 DArTseq markers on 508 discrete loci on the 19 chromosomes of *B. napus* (Raman R. et al., 2016) was used to determine the genetic basis of resistance to *L. maculans* pathotypes derived from mixed stubble. QTL analysis for resistance was performed using the three step procedures as outlined previously (Raman R. et al., 2016). A log-likelihood (LOD) score threshold of 2.0 was used to identify genomic regions associated with resistance to *L. maculans*.

(ii) Mapping of the *Rlm3* Locus in the AWDH Population.

Parental lines AG-Castle^∗^3 and Westar-10, along with *R* gene control lines (Westar, AV-Garnet, Surpass400, Caiman, ThunderTT, and Mustang), were characterized for their *Avr* profiles using a set of differential SSI of *L. maculans*. All 80 DH lines from AWDH, along with the parental lines were evaluated for the resistance or susceptibility to IBCN76 (*AvrLm1, AvrLm*3, *AvrLm*5, *AvrLm*6, *AvrLm*8, Avr*LepR1, AvrLepR3, AvrLepR4*), as described previously (Raman et al., 2012). Trays were arranged in a 4 row by 6 column array with replicates in a 2 row by 6 column array. Each tray held a row of 8 genotypes with each genotype being a column of seven plants. Genotypes were randomized to tray blocks and spatially arranged within trays using the DiGGer software (Coombes, 2002). GWAS associations linked with *Rlm3* were confirmed by selective genotyping of 23 resistant and 23 susceptible lines from the set of 80 DH derived from AWDH with 17,887 polymorphic DArTseq markers (Raman et al., 2014b). Of these, 13,296 DArTseq markers which had an overall call rate over 80% and reproducibility over 95% were used for linear marker regression in SVS package^2.^

### Statistical Analysis of Phenotyping Data

Disease scores collected from the different experiments were analyzed using linear mixed models with the statistical software package ASReml-R (Butler et al., 2009), which fits the linear mixed model using REML, within the R (R Development Core Team, 2014) computing environment. Internal infection on the crowns was recorded as percent infected area; logarithmic transformation was applied on data to normalize the residual variance and then used for QTL analysis. Predicted means were used to identify loci associated with resistance in the AWDH population using linear marker regression. The order of markers which were found to be significantly associated with resistance evaluated quantitatively (0–9 scores for cotyledon resistance and 0 to 100% for crown canker lesion) and ‘binned’ for resistance (‘A’ for resistance or ‘B’ for susceptibility), was determined in ‘Record’ (van Os et al., 2006) and with the FLAPJack software^3^.

### Prediction of Candidate Genes

A total of 128 *R* genes in *Arabidopsis thaliana* were retrieved from the TAIR^4^ and NCBI databases^5^. Physical map positions of 425 nucleotide binding site leucine rich repeat (NBS-LRR) encoding *R* gene homologs in *B. napus* were retrieved from the Genoscope^6^. These genes were predicted on the basis of Motif Alignment Search Tool E (≤-24) and tBLASTn and BLASTp values (1E-5) in *B. napus* sequence (Chalhoub et al., 2014). In order to obtain the physical map positions of *Arabidopsis R* genes, sequences were searched for their identities with the reference *B. napus* genome (Chalhoub et al., 2014) using BLAT function in *B. napus* Genome browser^2^. Only hits which showed high alignment scores (≥100) were considered to identify ‘candidate genes’ for resistance to *L. maculans*. We further searched potential candidate genes based on collinearity which were predicted in the vicinity of significant association that appeared both in GWAS and DH populations. LD decay that was estimated (*r*^2^ = 0.24 Mb) for the *B. napus* accessions was used for the identification of candidate genes (Raman H. et al., 2016).

## Results

### Genetic Variation for Resistance to *L. maculans*

**FIGURE 1:**
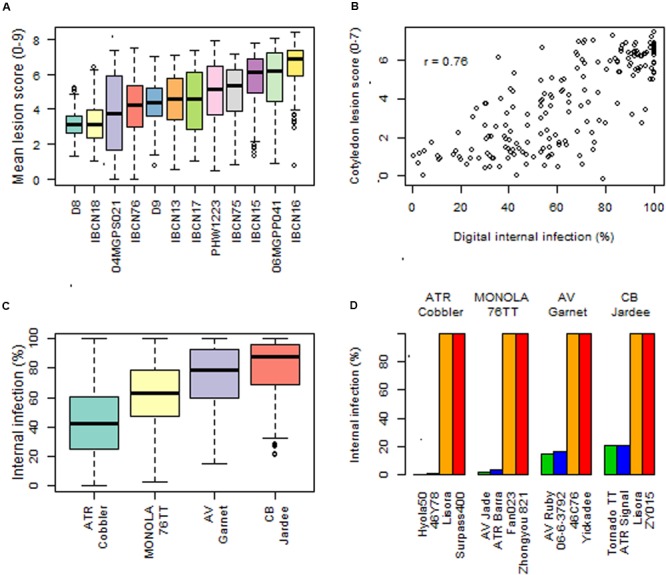
**Natural variation for resistance to *Leptosphaeria maculans*. (A)** Box-plots showing genetic variation for race-specific resistance to 12 SSIs in a diverse panel of 179 genotypes of *Brassica* under the glasshouse conditions. **(B)** Scatter graph showing relationship between cotyledon lesion and digital internal canker infection scores assessed under field conditions **(C)** Genetic variation for resistance evaluated at the adult plant stage using ascospore shower test in a diverse panel of 179 genotypes of canola under greenhouse conditions. Black bars represent median values and the colored boxes represent the range of variation in resistance and the error bars represent the outliers. **(D)** Promising canola genotypes which showed good level of resistance against pathotypes present on stubbles collected from the contemporary Australian canola varieties.

We determined the genetic resistance of 179 diverse accessions to 12 SSI of *L. maculans* at the seedling (cotyledon) stage. Across different experiments, we found extensive variation in resistance as lesion development ranged from 0 to 8.5 (**Figure 1A**; Supplementary Table S1). The majority of accessions were susceptible (cotyledon lesion scores >3) to different isolates (**Figure 1A**). Disease scores based on both visual and digitized score using isolate 04MGPS021 were highly positively correlated (*R*^2^ = 0.99, Supplementary Figure S1). To test the relationship between expression of resistance at both the seedling and adult plant stages, we compared cotyledon lesion and crown canker scores of 179 accessions of *B. napus* inoculated with 12 different isolates. Our results showed that resistance expressed at the cotyledon stage and in the crown canker was not always highly correlated; Pearson’s product-moment correlation coefficient ranged from 0.03 to 0.76 (Supplementary Table S2; **Figure 1B**), suggesting that some lines of the GWAS panel may have loci for adult plant and/or quantitative resistance.

In order to identify adult plant/quantitative resistance the GWAS panel was evaluated with four stubble sources collected from the commercial Australian canola cultivars AV-Garnet (*Rlm1*), ATR-Cobbler (*Rlm4, Rlm9*), CB-JardeeHT (*Rlm2*), and Monola76TT (*LepR3/Rlm1*). Our result showed that the majority of genotypes were susceptible to pathotypes especially present on stubble derived from AV-Garnet, CB-JardeeHT and Monola76TT (Supplementary Table S1; **Figure 1C**), suggesting that the *R* genes; *Rlm1, Rlm2, Rlm4, Rlm9*, and *LepR3* were no longer effective in conferring resistance to *L. maculans*. This study also revealed that some of the Australian cultivars carrying *R* genes, such as AV-Jade, ATR-Signal, AV-Ruby, 46Y78, TornadoTT, and ThunderTT were resistant to pathotypes present on at least three stubble sources (Supplementary Table S1; Figure ID). Several other accessions, such as Ag-Comet and StormTT, had a low level of disease. Our phenotyping experiments (SSI and ascospore shower test) showed that some genotypes in GWAS diversity panel may harbor quantitative resistance to Australian pathotypes of *L. maculans.*

### GWAS Detected ‘Known’ and ‘Novel’ Loci for Resistance to *L. maculans*

Genetic architecture of loci involved in resistance to *L. maculans* at both seedling and adult plant stage was dissected using GWAS by implementing a mixed model algorithm. The number of significant associations varied from 94 to 600 depending on the source of inoculum (600 with SSI, and 94 with pathotypes on four stubble sources (Supplementary Table S3). Manhattan plots of the significant associations detected with all 12 SSI are presented in Supplementary Figure S2.

(i) Natural Variation in Resistance to 12 Isolates of *L. maculans* at the Cotyledon Stage in a GWAS Panel.

The number of significant SNP associations varied from six with isolate D8 (*AvrLm5, AvrLm7, AvrLepR1, AvrLepr4*) to 237 with isolate PHW1223 (*AvrLm5, AvrLm6, AvrLm8, AvrLm9, AvrLepR1, AvrLepR3, AvrLepR4*) (Supplementary Table S3). Significant SNP loci accounted for 2.82 to 10.85% of total genetic variation for resistance to different isolates (Supplementary Table S3). For 04MGPS021, we detected 36 significant associations [at the genome-wide significance thresholds of a -log_10_(*p*) ≥ 3] on chromosomes A03, A04, A06, A07, A10, C02, C06, C08, and C09. Significant loci on homoeologous chromosomes A07/C06 accounted for a total of 35.31% of genetic variation in resistance (Supplementary Table S3).

Of 600 significant SNP associations for resistance to *L. maculans*, 76 were detected repeatedly across multiple isolates (Supplementary Table S3). These associations were detected with the SNPs that were localized at the same physical positions on the reference *B. napus* genome. In addition, several SNP markers which were in linkage disequilibrium (within 200 kb); estimated in a previous study (Raman H. et al., 2016) exhibited statistically significant association with resistance, suggesting that these markers could be used to trace introgression of favorable QTL alleles in canola breeding programs. Six markers (delimited with 5149488| 55:T > G, and 5030408| 14:C > T) localized within a 200 kb region on A07 (coordinates: 15778332–15923268 on the reference *B. napus* genome v 4.1), associated with *Rlm4* that was previously identified (Raman et al., 2012, 2014a) were detected across both subpopulations.

Our previous study showed that the GWAS panel used herein does have population structure which can highly affect the trait-marker associations for quantitative traits such as flowering time and response to vernalisation. In order to ascertain whether the population structure has any effect on detection of SNP associations (Raman H. et al., 2016) for qualitative traits, e.g., resistance to isolate 04MGPS021, we performed GWAS in individual subpopulations (I & II) and compared the GWAS results (Supplementary Figure S3). Subpopulations III and IV were very small in size (*n* = 8 to 11), therefore those were not suitable for GWAS. In subpopulation I (109 genotypes), 26 significant SNP associations (*p* value up to 1.54E^-6^) were detected on chromosomes A02, A07, A09, C02, C04, C06, and C09; of which eight were localized on homoeologous chromosomes A02/C02 and eleven on A07/C06. In subpopulation II (43 genotypes), 14 associations were detected on chromosomes A03, A05, A07, A10, C01, C03, C05, C06, and C07. Of them, six DArTSeq SNPs (one on A07, and five on C06) were present in LD estimated (*r*^2^ = 0.24 Mb) in *B. napus* accessions (Raman H. et al., 2016) used in this study. None of the markers on homoeologous chromosomes A02/C02; as detected in subpopulation-I, showed significant association with resistance in subpopulation-II, suggesting that subpopulation structure has profound effect on the identification of trait-marker associations.

(ii) Natural Variation in Resistance to *L. maculans* Evaluated with Ascospore Shower Test at the Adult Plant Stage.

**FIGURE 2:**
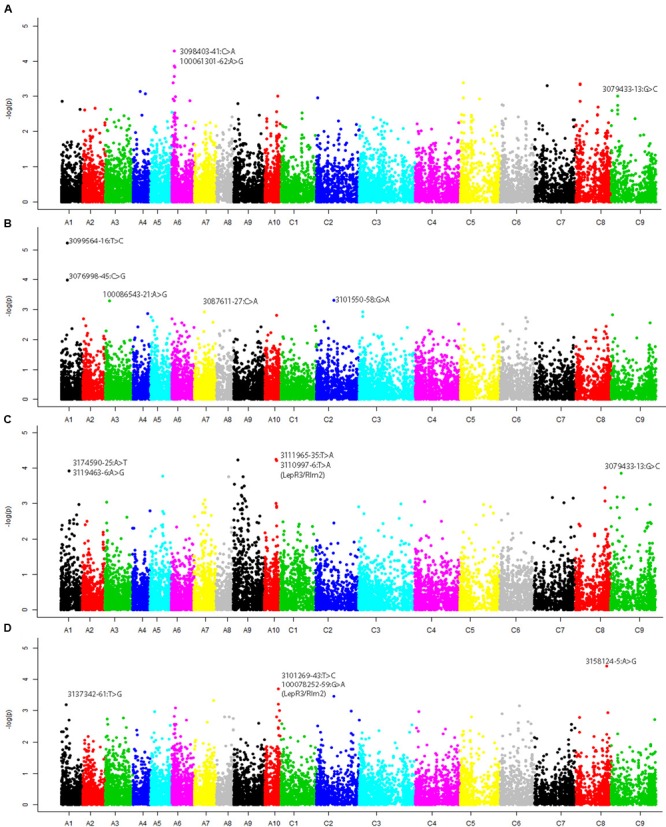
**Manhattan plots showing genome-wide *P* values for associations between SNP markers and resistance to *L. maculans* evaluated with ascospore shower test under greenhouse conditions in 179 canola accessions.** Stubble was collected from different canola varieties: **(A)** ATR-Cobbler, **(B)** AV-Garnet, **(C)** ATR-JardeeHT, and **(D)** Monola76TT. Different colors represent different chromosomes (A01–A10, C01–C9). Significant associations were tested at – log10(*p*) value of ≥3.

We revealed 94 statistically significant associations for resistance to *L. maculans*, scored as per cent internal canker infection on all *B. napus* chromosomes with the exception of A02 and C01 (Supplementary Table S3; **Figures 2A–D**). The number of SNPs for resistance detected with GWAS varied from 12 (with pathotypes present on stubble of AV-Garnet) to 45 (with pathotypes present on CB-JardeeHT stubble) (Supplementary Table S3). Significant associations for resistance to pathotypes on AV-Garnet stubble were detected on chromosomes A01, A03, A07, CO2, and unassembled contigs of the CnCn subgenome (ChrCnn_random) on the pseudomolecule of *B. napus* (Supplementary Table S3; **Figure 2B**). The maximum genotypic variance (10.5%) was explained by marker 3099564:16:T > C (-log10(*p*) = 5.95 × 10^-6^) mapped on chromosome A01. Forty five significant genomic regions for resistance to pathotypes on CB-JardeeHT stubble were identified on A01, A03, A05, A07, A08, A09, A10, C03, C04, C05, C07, C08, and C09 (**Figure 2C**). However, only four genomic regions on A06, and C09 revealed consistent associations with resistance to at least two stubble sources (Supplementary Table S3; **Figures 2C–D**).

Comparison between genome-wide associations detected for race-specific (SSI) and race non-specific (pathotypes from stubble sources) resistance revealed at least 25 genomic regions that appeared repeatedly (within LD; 200 kb region) on chromosomes A01, A03, A05/C05, A06, A10/C09, C02, C05, C08, and unassembled contigs on the An subgenome (ChrAnn_random) of the current genome assembly (Supplementary Table S3E). None of the consistent associations across experiments for resistance were detected on chromosomes A07, suggesting that the *R* genes; *Rlm1, Rlm3, Rlm4*, and *Rlm9* localized on A07 were in effective to provide resistance under field conditions (from where stubble sources were collected for ascospore shower test). Interestingly, a large number of significant associations (113) accounting for up to 10.84% phenotypic variation for resistance to SSI and pathotypes present on two stubble sources tested; CB-JardheeHT and AV-Garnet, were detected on homoeologous group 3 chromosomes, A03/C03. So far, no race-specific locus for resistance to *L. maculans* on these chromosomes has been reported in *B. napus, B. rapa*, and *B. oleracea*. In this study, we accounted for both population structure and kinship coefficients, therefore the likelihood of ‘false positive’ association is low, however, it needs further validation.

To assess whether resistance loci are clustered on any specific chromosomal regions, we compared the localisation of significant SNPs on the reference *B. napus* genome. We identified a higher frequency of significant SNP associations for resistance at seedling and adult plant stages on some chromosomes: A02 (2.73%), A03 (3.30%), A07 (9.88%), A10 (8.90%), and C03 (6.28%) (Supplementary Table S4). Only a small proportion of SNP markers (<2.2%) on A01/C01 were significantly associated with resistance to both seedling and adult plants stages, suggesting that a limited genetic variation occur at the detected loci in the accessions investigated.

### Validation of Significant GWAS SNPs via Linkage Mapping

(i) Validation of Linkage Between SNP markers and the Newly Identified *Rlm12* Gene in SAgSDH Population.

**FIGURE 3:**
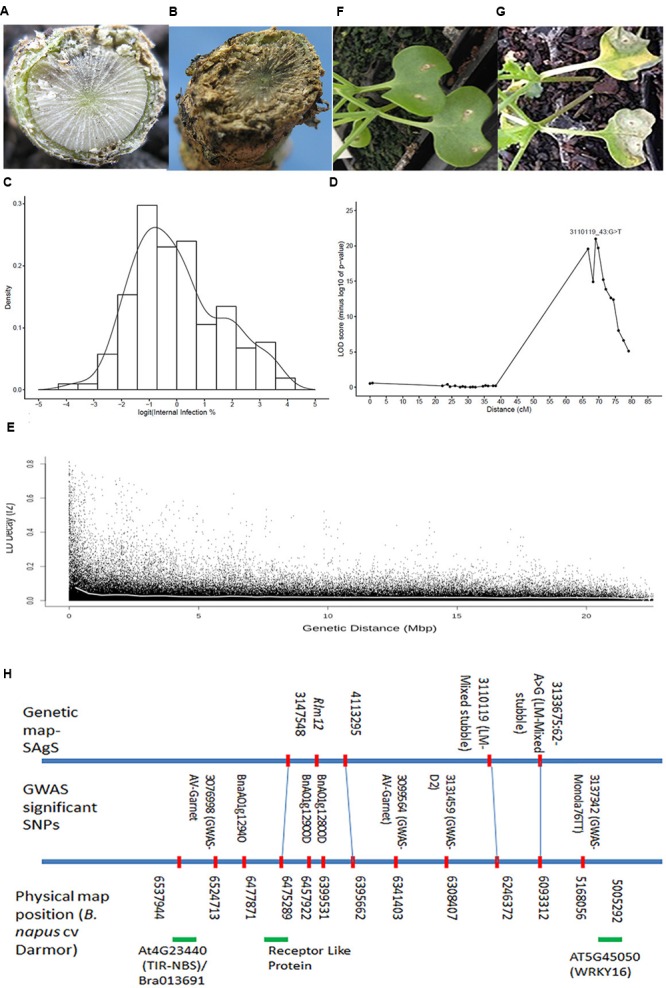
**Genetic and physical mapping of *Rlm12* locus on chromosome A01 in the SAgSDH population of *Brassica napus.*** Crowns of resistant *B. napus* cv. Ag-Spectrum **(A)** and susceptible *B. napus* cv. Skipton **(B)** showing canker due to infection of *L. maculans* pathotypes at the adult plant stage. **(C)** The frequency distribution of internal infection scores among DH lines from the SAgS cross. The population was scored for resistance at the physiological maturity. **(D)** A major quantitative trait locus (QTL) showing highly significant association with resistance to *L. maculans* pathotypes present on mixed stubble. **(E)** The average LD decay (*r*^2^) on chromosome A01 approach 0.029 when distance between SNPs was approximately 200 kb. The curve (shown in white) was drawn across SNPs markers using the non-linear regression model. **(F)** Cotyledons of Ag-Spectrum and Skipton **(G)** showing critical symptoms of *L. maculans* infection after 20 days of inoculation with a single spore isolate 06MGPP041. **(H)** Alignment of markers that showed significant association with resistance to *L. maculans* isolates, pathotypes present on different stubbles (ascospore shower screen in a GWAS panel and SAgSDH population) and the reference *B. napus* genome sequence cv. Darmor.

Genome-wide association analysis revealed eight significant SNP markers accounting for 5.23% to 10.50% of phenotypic variance on chromosome A01 in a GWAS panel when inoculated with two SSI; IBCN15 (*AvrLm5, AvrLm6, AvrLm8, AvrLepR1, AvrLepR3*, and *AvrLepR4*) and IBCN75 (*AvrLm1, AvrLm 5, AvrLm6, AvrLm8, AvrLepR1, AvrLepR3, AvrLepR4*), and stubble pathotypes derived from Monola76TT and AV-Garnet (Supplementary Table S3). These associations were further confirmed using the linkage mapping of the Skipton/Ag-Spectrum//Skipton (SAgSDH) population (146 lines). Significant genetic variation for resistance to *L. maculans* pathotypes was found among DH lines evaluated with pathotypes from mixed stubble using ascospore shower test (**Figures 3A,B**). Ag-Spectrum (inv.logit internal infection 58.1%) exhibited resistance as compared to Skipton (inv.logit internal infection 98.5%) (**Table 1**). The internal infection scores of DH lines ranged from 21.3 to 99.6% and mean internal infection (non-backtransformed mean) was 72%. The DH lines showed continuous distribution for multigenic resistance (**Figure 3C**), unlike the AWDH population. Most of the natural variation was genetically controlled as the broad sense heritability was high (67%).

**Table 1:** Genetic variation for resistance to pathotypes present on mixed stubble collected from the commercial Australian canola cultivars AV-Garnet, ATR-Cobbler, CB-JardeeHT, and Monola76TT.

Genotype	Predicted. value.logit	Predicted. value.invlogit	Standard. error.logit
Ag-Spectrum	0.14	58.10	2.05
DH lines	1.81	87.36	0.47
Skipton	4.11	98.55	2.05

*“Data was analyzed using logistic transformationBacktransformed” (invlogit) values also provided as a reference.*

QTL analysis of the DH lines revealed five putative genomic regions for resistance to *L. maculans*; of which one highly significant locus [-log10(*p*) = > 26] was identified on chromosome A01 (**Table 2**; **Figure 3D**). This major QTL could explain 24.6% of the genotypic variance for resistance. Ag-Spectrum allele increased the resistance to *L. maculans* pathotypes as expected from phenotypic disease scores (**Table 2**). Four minor QTL (LOD score ≤ 2.3) were identified on chromosomes A02, A06, A07, and C08 (**Table 2**). To confirm the presence of QTL detected with WGAIM approach (Raman R. et al., 2016), linear marker regression was conducted in the SVS package using DArTseq markers and predicted means of DH lines. SNPs 3133675_62:A > G and 3110119_43:G > T on chromosome A01 showed highly significant association [-log10(*p*) = 32 to 32.8; Bonferroni *P* values = 4.52 × 10^-32^ to 8.40 × 10^-31^) with resistance to *L. maculans* pathotypes (Supplementary Table S5). In order to determine whether genetic variation at the QTL on A01 is due to a major locus, we ‘Mendelise’ quantitative disease scores into resistant and susceptible bins and performed linkage mapping. The *QRlm.wwai-A01* was mapped as a single *R* locus and designated as *Rlm12* (**Figure 3H**). This newly identified locus was delimited with 3147548–4113295 marker interval on the genetic linkage map of SAgSDH population (Raman R. et al., 2016).

**Table 2:** Quantitative trait locus (QTL) associated with adult plant resistance (APR) to *Leptosphaeria maculans* pathotypes present on mixed stubble sources.

Marker	Chromosome	Genetic map position	Additive Effect	P value	LOD	*R*^2^ (%)
3110119_43:G > T	A01	69.0	-3.31	4.14e-27	26.38	24.57
3145775	A02	36.81	0.70	1.50e-02	1.82	1.42
3154085	A06	0	-0.77	5.51e-03	2.26	1.82
3117277	A07	39.59	-0.78	7.94e-03	2.10	1.69
3084361	C08	27.16	0.75	1.05e-02	1.98	1.61

*Resistance was tested using ascospore shower test (Huang et al., 2006b).*

In order to verify whether the genomic region on A01 detected in the GWAS diversity panel (this study) and SAgSDH population evaluated under ascospore shower test, is the same as detected previously with isolate 06MGPP041 in a SASDH population (Raman et al., 2012), we re-evaluated the SASDH population for cotyledon resistance against 06MGPP041 under the glasshouse conditions. Cotyledons of Ag-Spectrum showed classical hypersensitive response on infection with the isolate 06MGPP041, consistent with the ETI mechanism established for *R* genes (**Figures 3F,G**). Utilizing a linkage map previously constructed for SASDH population (Raman et al., 2014b), resistance to *L. maculans* isolate 06MGPP041 was remapped using a cotyledon lesion scores on chromosome A01 near Infinium 6K SNP markers UQnapus0918/UQnapus3827 [–log10(*p*) > 2.5, favorable resistance allele: Ag-Spectrum].

(ii) Validation of Linkage Between SNP Markers and the *Rlm3* gene in DH Population from AG-Castle^∗^3/Westar-10 (AWDH).

**FIGURE 4:**
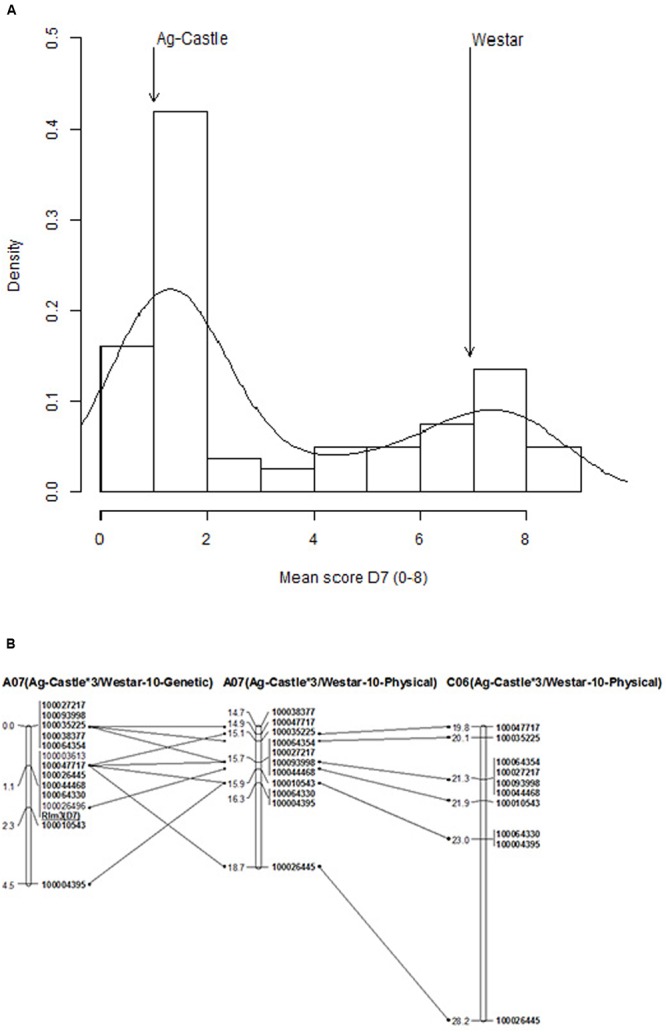
**Genetic mapping of *Rlm3* locus for resistance to blackleg in Ag-Castle^∗^3/Westar-10. (A)** The frequency distribution of cotyledon lesion scores among DH lines from Ag-Castle^∗^3/Westar-10. **(B)** Physical mapping of *Rlm3* gene for resistance to *L. maculans* on A07 and C06 (details are given in Supplementary Table S3). The marker order is shown on the *right hand side*, while the genetic and physical distances (x1Mbp) are on the *left hand side*.

The presence of the *Rlm3* in AG-Castle was validated using a differential set of isolates (Supplementary Table S6). Isolate IBCN76 was avirulent toward *Rlm3* and produced resistant reaction (1 on a 0 to 9 scale) on AG-Castle^∗^3 compared to susceptible reaction (7 on a 0 to 9 scale) on Westar-10. The disease scores on DH lines exhibited the hallmark bimodal distribution (**Figure 4A**), suggesting that most likely a single gene controls resistance to isolate IBCN76. The broad sense heritability was high (90.3%), suggesting that most of the phenotypic variation for resistance is genetically controlled.

DArTseq marker analysis of the AWDH population revealed a total of high quality 13,296 polymorphisms which showed segregation among 46 selected lines (Supplementary Table S7a). Of the 180 significant associations [-log10(*p*) ≥ 3], five DArTseq markers 100047717-11:C > G, 100026445-14:C > T, 100044468-36:C > T, 100064330-45:C > T and 100003613-47:G > C showed highly statistically significant association [-log10(*p*) = 19.68] with resistance to isolate IBCN76 (Supplementary Table S7b). In order to determine the relative position of the *Rlm3* locus to the highly significant SNPs (revealed after correcting *P* value calculated with Bonferroni test = 3.76 × 10^-6^), we binned disease scores into two categories, resistant and susceptible. The *Rlm3* locus from AG-Castle was mapped on chromosome A07, and showed a complete linkage with a suite of six markers (**Figure 4B**). Seven DArTseq markers which showed linkage with *Rlm3* also revealed homoeology with a genomic region spanning 8.41-Mbp on the physical genomic map of chromosome C06 of the reference sequence of cv. Darmor-*bzh* (**Figure 4**), suggesting that a homoeologous genomic region on A07/C06 may control resistance to blackleg. Two SNP markers 100003613 and 100026496 located on chromosome A07_random contig, were mapped on A07 and showed tight linkage with *Rlm3* in the AWDH population (Supplementary Table S3).

(iii) Validation of Linkage Between SNP markers and Known *Rlm1, Rlm4*, and *LepR3* Genes in DH Populations.

**FIGURE 5:**
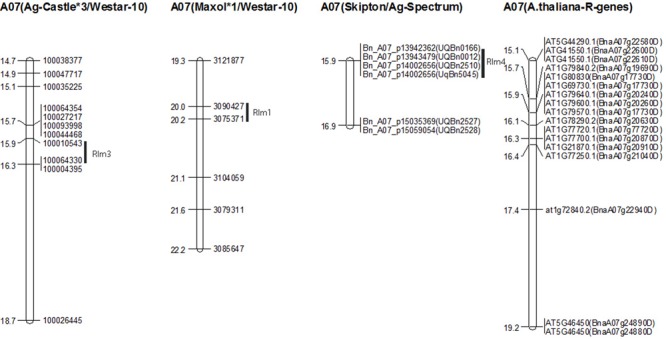
**Physical mapping of *Rlm1, Rlm3*, and *Rlm4* genes of *B. napus* associated with resistance to *L. maculans* and disease resistance genes of *Arabidopsis* on the sequenced genome of *B. napus* cv Darmor.** The marker order is shown on the *right hand side*, while the physical distances (x1Mb) are on the *left hand side*. The markers labeled with prefix ‘Bn_A07_p’ and UQBn’ represent to 6K and 60K Infinium SNP markers, respectively. *Arabidopsis* disease resistance genes are labeled as ‘At’. Genes with the prefix ‘Bna’ represent to *B. napus* predicted *R* genes.

In order to validate whether the genomic regions associated with resistance to *L. maculans* delineated in this GWAS are indeed ‘true’, we aligned adjacent sequence based DArTseq and 6K Infinium SNP markers (Dalton-Morgan et al., 2014) flanking QTL regions associated with resistance in four DH mapping populations derived from crosses AG-Castle^∗^3/Westar-10 (this study), BLN2762/Surpass400 (Raman R. et al., unpublished), Maxol^∗^1/Westar-10 (Raman R. et al., 2013), and Skipton/Ag-Spectrum (Raman et al., 2012; Tollenaere et al., 2012), with the published *B. napus* genome sequence (Chalhoub et al., 2014). Infinium SNP markers Bn_A07_p13943479 (UQBn0012), Bn_A07_p14002656 (UQBn2510 and UqBn5045), and Bn_A07_p15059054 (UQBn2528) closely linked with the *Rlm4* locus for resistance to isolate 04MGPS021 in a Skipton/Ag-Spectrum (SASDH) population were localized on A07 (**Figure 5**) within 100 kb (88-kbp to 93-kbp) from the significant GWAS SNPs (Supplementary Table S8). This suggested that genome-wide associations identified in this study are indeed robust and valid. Likewise, GWAS associations were also verified for *Rlm1* locus in DH populations derived from Maxol^∗^1/Westar-10 (MWDH) (within 241 kb), and *LepR3* gene (∼within 400 kb) in the BLN2762/Surpass400 (BSDH) (Supplementary Table S8; **Figure 5**). The genetic linkage map of MWDH population (A07) was collinear, except the DArTseq marker 3077579 to the physical map of *B. napus* (Supplementary Figure S4), suggesting that DArTseq markers were anchored correctly on their respective genetic position.

### Prediction of Resistance Loci Using GWAS SNP

In order to verify whether significant GWAS SNPs accurately predict the known *Rlm1, Rlm3, Rlm4*, and *LepR3/Rlm2* and the proposed *Rlm12* loci for resistance, we filtered marker alleles in Microsoft excel and compared their allelic profiles with cotyledon lesion (mean) scores from differential set of isolates (Supplementary Table S9). Our results reiterated that SNP haplotypes were consistent in detecting resistance loci in Australian canola varieties. Several resistance sources could be tracked accurately, consistent with their breeding history. For example, the haplotype based on two GWAS SNPs (3100490| 56:A > T and 3132403| 42:G > T) linked with the *Rlm4* locus on A07 enabled us to predict *Rlm4* mediated resistance in several canola varieties such as 46C76, AV-Sapphire, BLN2762, BLN3347, CB-Telfer, Monty, Tarcoola, TornadoTT, ThunderTT, Scoop, and Skipton. All these varieties had cotyledon lesion scores ≤3.5 upon inoculation with IBCN17 and IBCN18 differential isolates (Supplementary Table S1). Prediction of *Rlm4* mediated resistance in these varieties is also consistent with previous studies (Marcroft et al., 2012; Raman et al., 2012). Similarly, a suite of five markers enabled us to track the *Rlm12* mediated adult plant resistance (APR) in Ag-Spectrum, Ag-Comet, ATR-Signal, AV-Ruby, HurricaneTT, TornadoTT, and ThunderTT. Pedigree analysis revealed that several of these varieties (Ag-Comet. ATR-Signal, AV-Ruby, ThunderTT, and TornadoTT) are genetically related ^7^ (Raman et al., 2014b).

### GWAS Identifies ‘*Rlm*’ Genes for Resistance to *L. maculans* in Canola

In order to identify potential candidate genes involved in the qualitative and quantitative resistance to *L. maculans*, we compared 78 highly significant SNP associations after applying Bonferroni correction [*p* < 2.68 × 10^-5^ or -log10(*p*) > 4.57] for resistance detected in the GWAS diversity panel phenotyped under the glasshouse (cotyledon test) and greenhouse conditions (ascospore shower screen) with the sequenced *Rlm2*, and *LepR3*, 425 *R gene* homologs of *B. napus* (Chalhoub et al., 2014) and other 126 *R* genes of *A. thaliana* (Supplementary Table S3E). We further searched potential candidate genes based on collinearity which were predicted in the vicinity of significant association that appeared both in GWAS and DH populations. Of highly significantly associated SNP loci for resistance to *L. maculans* pathotypes, 32 were localized within 200 kb from *R* genes of *A. thaliana* (**Table 3**; Supplementary Table S3E). Besides, a total of 48 NBS-LRR homologs of *B. napus* (Chalhoub et al., 2014) could be localized within 200 kb genomic region of SNP associations (data not shown); of them four were located on A01, A05, and A07 chromosomes within 150 kb from highly significant SNP associations (Supplementary Table S3E; **Figure 3E**). Two *B. napus R* gene homologs were localized within a 10 kb region of GWAS associations on A03 (AT2G14080.1) and A07 (AT1G72890.2) encoding R protein having TIR-NBS-LRR motifs (Supplementary Table S3). Significant SNPs for resistance against different isolates and stubble sources were detected within the genomic region of 13.4 to 14.4 Mbp on chromosome A10 representing to *LepR3* and *Rlm2* (BnaA10g20720D/AT2G15042.1) genes (Supplementary Table S3). The AT2G15042 gene encoding an LRR family protein on chromosome 2 of *A. thaliana* (6510165–6512335) was localized within 1.8 kb from BnaA10g20720D, suggesting that *Arabidopsis* based collinearity could be utilized to identify candidate genes for resistance to *L. maculans* in canola.

**Table 3:** Genome-wide significant SNP associated with variation in resistance to *L. maculans* at the cotyledon stage (cs).

Isolate/stubble source used for host– pathogene interaction	SNP	Chr	Physical Position on *Brassica napus* genome	-*log10(P)* value	Var (*R*^2^)	Candidate gene (‘At/Bna’)	Physical Dist. (Mbp) from Position of candidate gene in reference Darmor psedomolecule	Other significant SNP found within 200 Kbp region for
D10 (PHW1223)-CS	3083011| 51:C > T	A02	10006975	3.4222	4.195381	AT1G54470.2	3662	Nil
D10 (PHW1223) -CS	3084495| 49:A > G	A02	17073426	-4.48563	5.816851	AT5G44870.1/ BnaA02g23800D AT5G44870.1	6779	Nil
D10 (PHW1223)-CS	5052687| 29:A > C	A03	1588113	-4.98757	6.60727	AT3G44410.1/ BnaA03g03260D	403	D2 (IBCN15), D6 (IBCN75), D9
D6 (IBCN75) -CS	4337290| 27:A > T	A06	16217358	-3.05173	4.669065	AT5G64140.1	3591	Nil
D10 (PHW1223) -CS	5048140| 18:C > G	A06	19634608	3.0	3.559926	AT3G27450.1	2789	Nil
Monola76TT	3101269| 43:T > C	A10	14658053	-3.70658	6.823456	AT2G14080.1	1989	Nil
D3 (IBCN16) -CS	3133437| 61:T > C	C07	33434383	2.93	5.36589	AT3G27450.1	3798	NIl
D10 (PHW1223) -CS	3079433|F| 0-13:G > C-13:G > C	C09	6973984	-3.06982	3.675336	AT2G20310.1	2436	^∗^CB-JardheeHT, ^∗^ATR-Cobbler
04MGPS021	4109828| 18:T > C	C09	40309142	3.009	5.292392	AT3G04210.1	9664	

*Only markers which were localized within 10 Kb from disease resistance genes of *Arabidopsis* (with At prefix) and *B. napus* (with Bna prefix) are shown here. Details are given in Supplementary Table 3. ^∗^collocated on the same position of *Brassica napus* genome*

Two genomic regions harboring *Rlm1, Rlm3*, and *Rlm4* genes; delimited with coordinates 14.74 Mbp to 17.02 Mbp and 19.27 Mbp to 23.3 Mbp of A07 in the *B. napus* reference genome contained significant SNPs associated with resistance to IBCN15, IBCN17, IBCN75, IBCN76, D9, PHW1223, 04MGPS021 in GWAS, and DH populations derived from SASDH, AWDH, MWDH, and BSDH (Supplementary Table S3). This genomic region represents to collinear region on *Arabidopsis* chromosome 1 for resistance to *L. maculans* gene *AtRlm1* (Mayerhofer et al., 2005) and corresponds to *Rlm1, Rlm3, Rlm4*, and *Rlm9* genes in *B. napus* (Delourme et al., 2004; Parkin et al., 2005). Several *R* genes encoding TIR-NB-LRR and CC-NB-LRR domains (BnaA07g17000D, BnaA07g22940D, BnaA07g26100D BnaA07g30480D BnaA07g34770D BnaA07g35000D, and BnaA07g35000D), *ROP3, PEN3, GST, SPL6*, RbohF, protein kinase, and pathogenesis related proteins (AT5G46450.1 on *At* 5 chromosome) were also localized in both genomic intervals.

*RLM1col* gene (AT1G64070.1) for resistance to *L. maculans* in *A. thaliana*, was located approximately 150 kb from GWAS SNP, 3083208| 11:T > A detected for resistance to PHW1223 on chromosome C03. Physical mapping of significant SNPs on chromosome A01 and *R* genes of *A. thaliana* and *B. napus* on the reference sequenced genome of *B. napus* revealed that a genomic region spanning 1.3 Mb delimited with WRKY16 (AT5G45050/BnaC03g08660D) and TIR-NBS involved in signal transduction, apoptosis, and innate immune response in *Arabidopsis* (AT4G23440 on chromosome 4), *B. rapa* gene (Bra013691), and *B. napus* receptor like protein (BnaA01g12940D) is associated with resistance to *L. maculans* isolate IBCN15 [-log10(*p*) ≤ 3], stubble pathotypes derived from Monola76TT, AV-Garnet and mixed stubble in a SAgS DH population (**Figure 3H**). This suggested that utilization of GWAS and linkage mapping approaches in parallel enabled us to identify a ‘potential’ candidate gene underlying *Rlm12* mediated resistance to *L. maculans*.

## Discussion

Identification of ‘durable’ and novel alleles for qualitative and quantitative resistance to the devastating blackleg disease is critical for sustainability of canola industry. GWAS and linkage analyses enabled us to identify and validate statistically significant marker loci for known *R* genes to *L. maculans* (*Rlm1, Rlm3, Rlm4*, and *LepR3*). This has been possible due to the comprehensive analysis of 179 *B. napus* diverse lines, 12 SSI, and mixed pathotypes (present on the four different stubble sources) interactions, and a good understanding of chromosomal location of qualitative *R* loci in *B. napus.* In addition, we identified several new loci for resistance (Supplementary Table S3); one of them, *Rlm12* was identified in a GWAS panel and validated in SAgSDH population. Our linkage mapping results suggested that *Rlm12* is a major gene having large allelic effects and also provides APR. Previously, QTL for field resistance accounting for *R*^2^ = 14 to 24.6% on chromosome A01 were reported in Australian DH populations derived from AV-Sapphire/Westar-10, Caiman/Westar-10 and Camberra/Westar-10, and Skipton/Ag-Spectrum (Kaur et al., 2009; Raman et al., 2012). In addition, Raman et al. (2012) reported a QTL; *QRlm.wwai-A1* (LOD score: 3.0) accounting for 22.8% of genetic variance on A01 for resistance at seedling stage with the isolate 04MGPP041. In this study, we showed that parental lines of a SAgSDH population exhibit a classical hypersensitive response on inoculation with 06MGPP0P41 (**Figures 3G,H**), as observed for *R* genes. Detection of low level of genetic variation for resistance at the seedling stage (Raman et al., 2012) and mapping of a major locus for resistance at the adult plant stage on A01 (this study) suggest that *Rlm12* may represent an APR locus. Marker haplotypes showed that several accessions such as Ag-Spectrum, AV-Sapphire, AV-Ruby and ATR-Signal may carry the *Rlm12* locus for resistance and may have provided resistance against different pathotypes under field conditions but due to the absence of a differential isolate set, this may have remained undetected in the Australian canola germplasm (Supplementary Table S3). Evidence of APR was also obtained from the phenotypic data obtained from SSI, as the cotyledon lesion score and internal infection was poorly correlated (Supplementary Table S2). Resistance expressed at the adult plants stage could be due to race non-specific APR genes (e.g., *Rlm12*), race-specific *R* genes (Delourme et al., 2004, 2011; Raman et al., 2012; Larkan et al., 2013, 2015) and race non-specific quantitative resistance (Delourme et al., 2006; Rimmer, 2006; Huang et al., 2009). This study revealed that some of the Australian cultivars were either resistant to pathotypes present on at least three stubble sources or had a low level of disease (**Figure 1D**, Supplementary Table S1). These specific varieties may provide a useful resource for genetic improvement of quantitative resistance to *L. maculans.*

We established that several GWAS SNP associations are indeed associated with resistance to *L. maculans*; however, some of them need to be validated in relevant populations. The genomic regions (SNP associations) on A02, A06, A07, and A10 may represent well defined race-specific *R* genes: *LepR1* (on A02); *LepR4* (on A06); *Rlm1, Rlm3, Rlm4, Rlm7*, and *Rlm9* (on A07), and *LepR3*/*Rlm2* on A10 controlling natural variation for resistance to *L. maculans* (Delourme et al., 2004, 2011; Raman et al., 2013b; Yu et al., 2013; Larkan et al., 2015). Our results also showed that highly significant associations for resistance can be identified in a small set of genotypes (subpopulation-II and AWDH population). Several GWAS SNPs for qualitative (cotyledon) and/or quantitative (crown canker) resistance were localized in homoeologous regions, particular on linkage groups A01/C01, A02/C02 A03/C03 A05/C05, and A07/C06. These regions may either represent functionally redundant loci or involved in increased allelic diversity of the genes controlling resistance to blackleg (Fopa Fomeju et al., 2014). Genome-wide associations detected in a GWAS panel on chromosomes A03/C03 may represent novel alleles or their interactions with the known R loci. It is also possible that resistance to *L. maculans* is confounded with other phenological attributes such as flowering time and plant maturity. The GWAS panel used herein possessed diverse accessions representing winter-, spring-, and semi-winter types (Raman H. et al., 2016) and included derivatives from *B. napus*/*B. juncea* (Roy, 1984) which may have resistance genes introgressed from B genome. Chromosome B3 in *B. nigra/B. juncea*, which is orthologous to *B. rapa* and *B. napus* chromosome A03, has been implicated in conferring blackleg resistance. We have not validated whether the statistical significant associations identified on A03/C03 either represent to (i) qualitative/quantitative resistance to *L. maculans* (Supplementary Table S3, (Fopa Fomeju et al., 2014), or due to (ii) pleotropic traits involved in phenological components. Comparative mapping showed that At3g15190 and *BnaA03g03260D* (AT2G14080.1, disease resistance protein (TIR-NBS-LRR class) genes could be localized in the same QTL region identified for resistance on A03/C03 in this study. At3g15190 gene also showed sequence identities with chromosomes B01/A01/C01, and B05/A05 (Panjabi et al., 2008). Large numbers of associations detected for specific isolates (e.g., PHW1223) may also be due to the genetic network involved in resistance to *L. maculans* to different *Avr* genes present. For example, SOBIR1 which was physically mapped on A03/C03 and A04 chromosomes (Supplementary Table S3) is suggested to interact with LRR-RLP (*LepR3*) in conferring resistance (Larkan et al., 2013; Liebrand et al., 2014).

The completely sequenced and annotated genomes of *A. thaliana* and *B. napus* proved to be useful to unravel candidate genes in *B. napus;* several paralogs for resistance to *L. maculans* were identified. For example, *Rlm1, Rlm3*, and *Rlm4* were mapped in the vicinity of R genes such as *ABCG36* and *RAC-LIKE 1*, and protein kinase genes (Supplementary Table S3, **Figure 5**). Previously, the same gene (At1g64070) has been shown to confer resistance to *L. maculans* in *A. thaliana*. *R* genes encoding CC-LRR-NBS, TIR-LRR-NBS, receptor-like kinase, receptor-like protein, transmembrane and PTO domains localized in the vicinity of significant SNP associations (Supplementary Table S3) have been implicated in signal perception and transduction, apoptosis, adaptive and innate immune response to positively enhance resistance function of disease resistance (Bent et al., 1994; Meyers et al., 2003; Mun et al., 2009; Yu et al., 2014; Larkan et al., 2015). Genome wide analyses of *B. napus* and *B. rapa* genomes revealed that *R* gene analogs are frequent (Wang et al., 2011; Chalhoub et al., 2014). We found that these genes were often localized close to the significant SNPs that we have identified in GWAS and DH populations in this study. It was interesting to note that some of the key genes such as *LepR3/Rlm2* were not the closest (within LD: 200 kb) from the SNPs detected with GWAS. This may be due to lack of marker polymorphism in the mapping panels, moderate genome-wide coverage of markers and low frequency of informative alleles (associated with resistance to *L. maculans*).

## Conclusion

GWAS and linkage analysis approaches delineated both known and unknown (new) genomic regions that control the resistance to *L. maculans* in *B. napus* and resolve genomic regions to a single gene level. We were able to identify *Arabidopsis thaliana*/*B. napus* genes that were present near loci exhibiting natural variation for resistance to *L. maculans* in a GWAS panel. Validation of significant GWAS markers associated with both race-specific (detected with multiple isolates) and race non-specific loci (naturally occurring pathotypes present on different stubbles) in DH populations suggested that these loci could be manipulated to enhance background level and durability of resistance in *B. napus* germplasm.

## Author Contributions

HR and RR conceived and designed the study; NC and SD developed experimental design and analyzed phenotypic data; RR, HR, and KL phenotyped populations for blackleg resistance using SSI; SM, RR, and HR conducted ascospore shower test experiments on GWAS panel; SM, DB, and PS phenotyped SAgSDH population using ascospore test and provided seed of AWDH population; HR, RR, AK, and JS conducted molecular and association analyses, and determined physical locations of SNP and candidate genes of *A. thaliana*; JB and DE provided physical locations of *R* genes in the *B. napus* genome; HR and RR interpreted the data, prepared the manuscript and supervised the whole study; all authors reviewed and edited the manuscript.

## Conflict of Interest Statement

DArT P/L (authors JS and AK) (Canberra, Australia) is a genotyping company and may benefit from providing genotyping service of DArTseq markers to the Brassica R&D community. The other authors declare that the research was conducted in the absence of any commercial or financial relationships that could be construed as a potential conflict of interest.

## Footnotes

1 https://imagej.nih.gov/ij/

2 http://www.goldenhelix.com/

3 https://ics.hutton.ac.uk/flapjack/

4 http://www.arabidopsis.org/

5 http://www.ncbi.nlm.nih.gov/Genomes/

6 http://www.genoscope.cns.fr/brassicanapus/

7 http://pericles.ipaustralia.gov.au/pbr_db/
