# Supplementary material for: Genome-wide Association Study Identifies New Loci for Resistance to Leptosphaeria maculans in Canola
Source: Front Plant Sci. 2016 Oct 24;7:1513. doi: 10.3389/fpls.2016.01513 (PMC5075532; doi:10.3389/fpls.2016.01513)

Figure S1: Pair plots showing correlations in genetic variation for resistance to *L. maculans* evaluated using cotyledon test (lesion score), per cent internal canker infection (internal infection) assessed visually and from digital images (digital scores) in a GWAS panel.

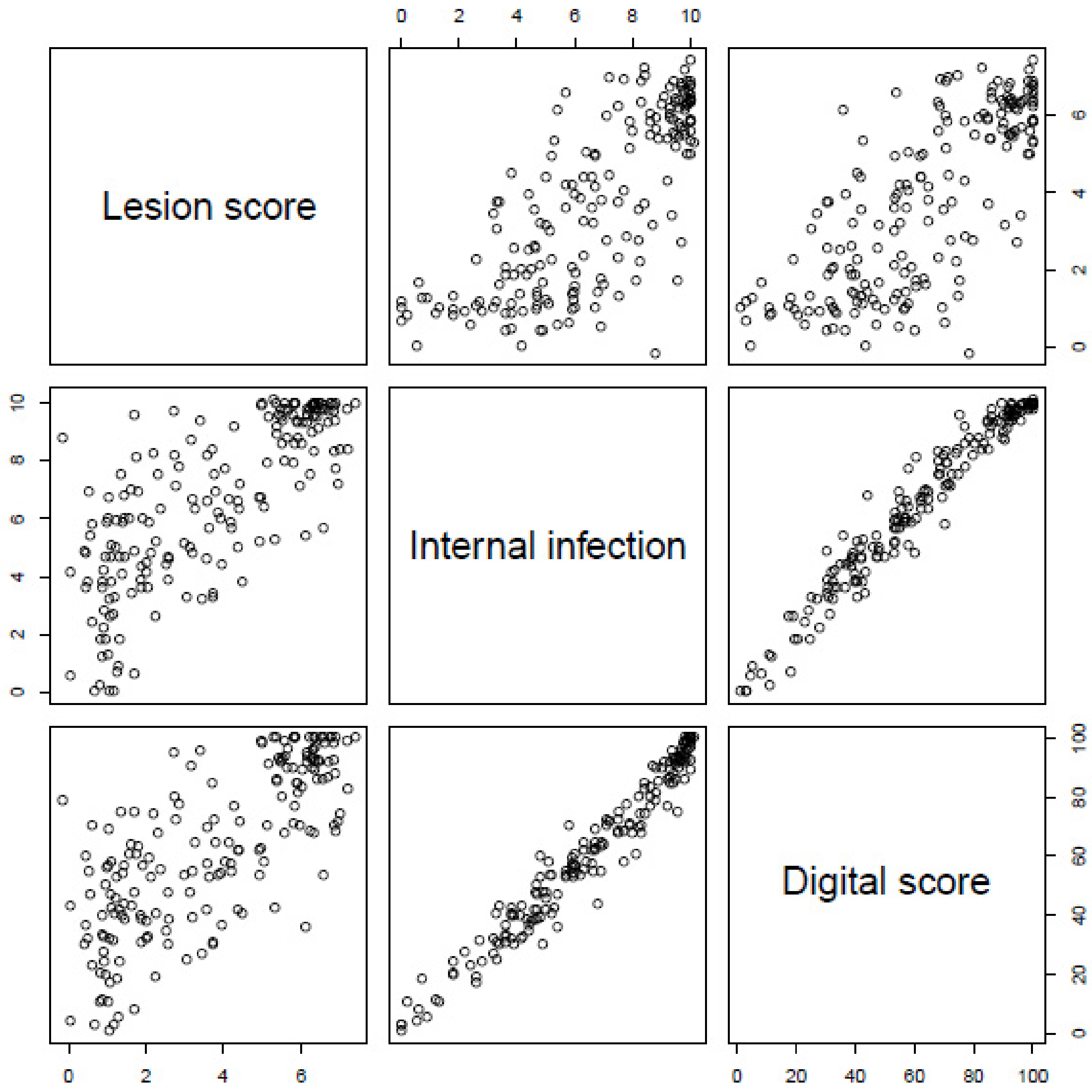

Supplement: Supplementary file 10 [file Image_1.pdf]
