# Supplementary material for: Genome-wide Association Study Identifies New Loci for Resistance to Leptosphaeria maculans in Canola
Source: Front Plant Sci. 2016 Oct 24;7:1513. doi: 10.3389/fpls.2016.01513 (PMC5075532; doi:10.3389/fpls.2016.01513)

Figure S2: Manhattan plots showing genome-wide *P* values for associations between SNP markers and resistance to *L. maculans* evaluated with 12 single spore isolates (04MGPS021, 06MGPP041, D8, D9, IBCN13, IBCN15, IBCN16, IBCN17, IBCN18, IBCN75, IBCN76 and PHW1223) and ascospore shower test under greenhouse conditions in 179 canola accessions. Significant associations were tested at - log10(*p*) value of ≥ 3.

D1(IBCN13)

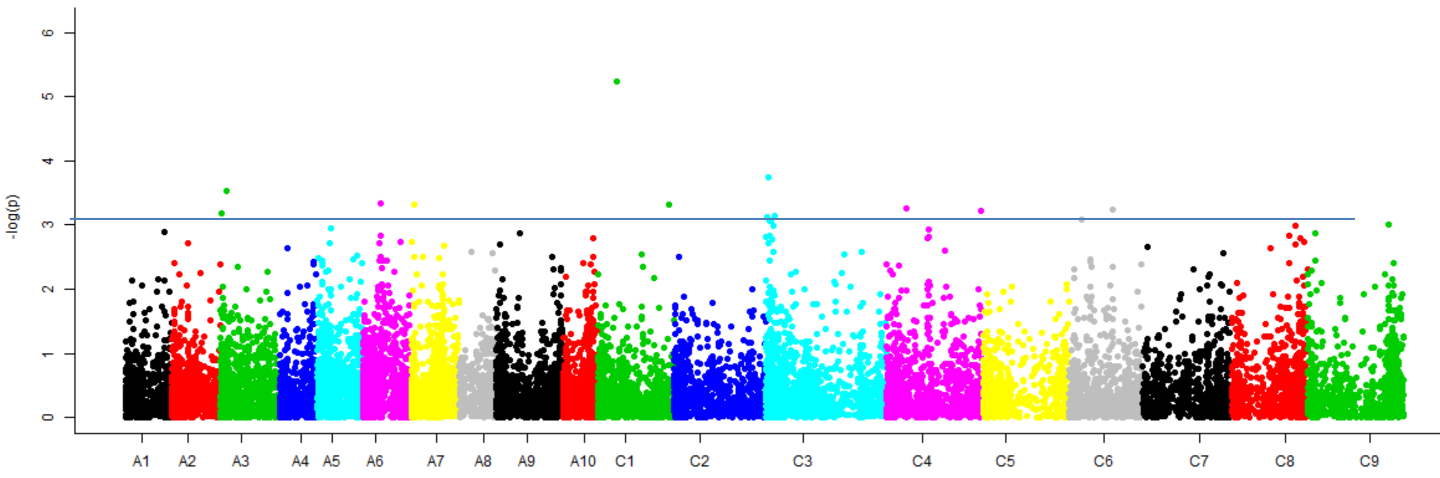

D2 (IBCN15)

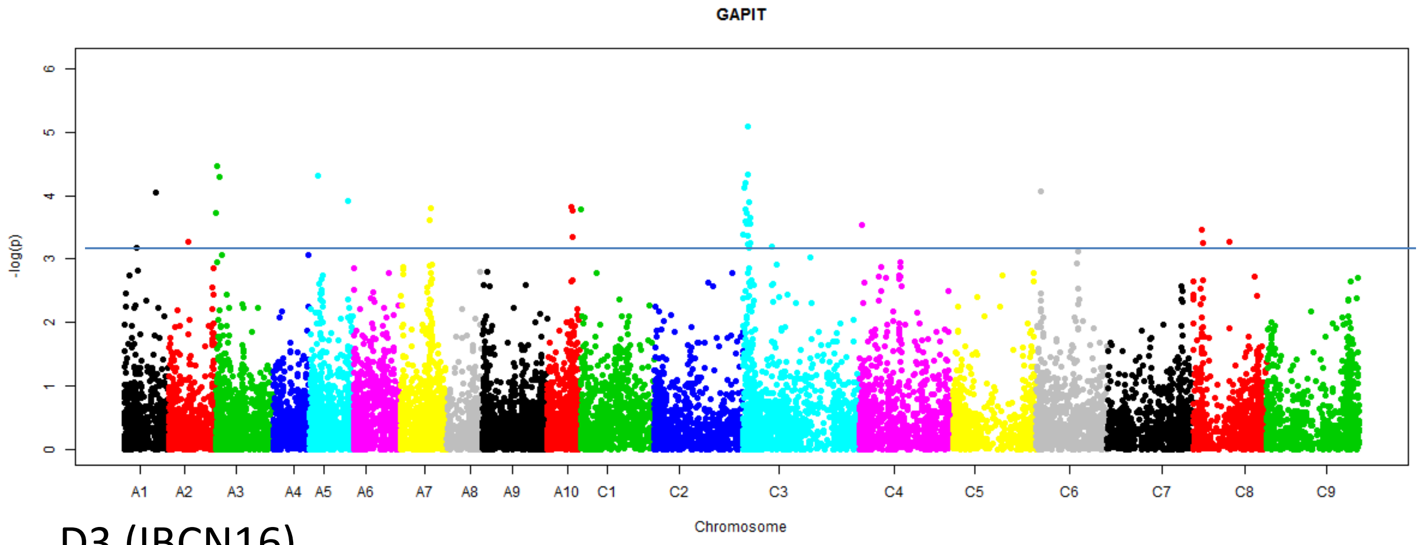

D3 (IBCN16)

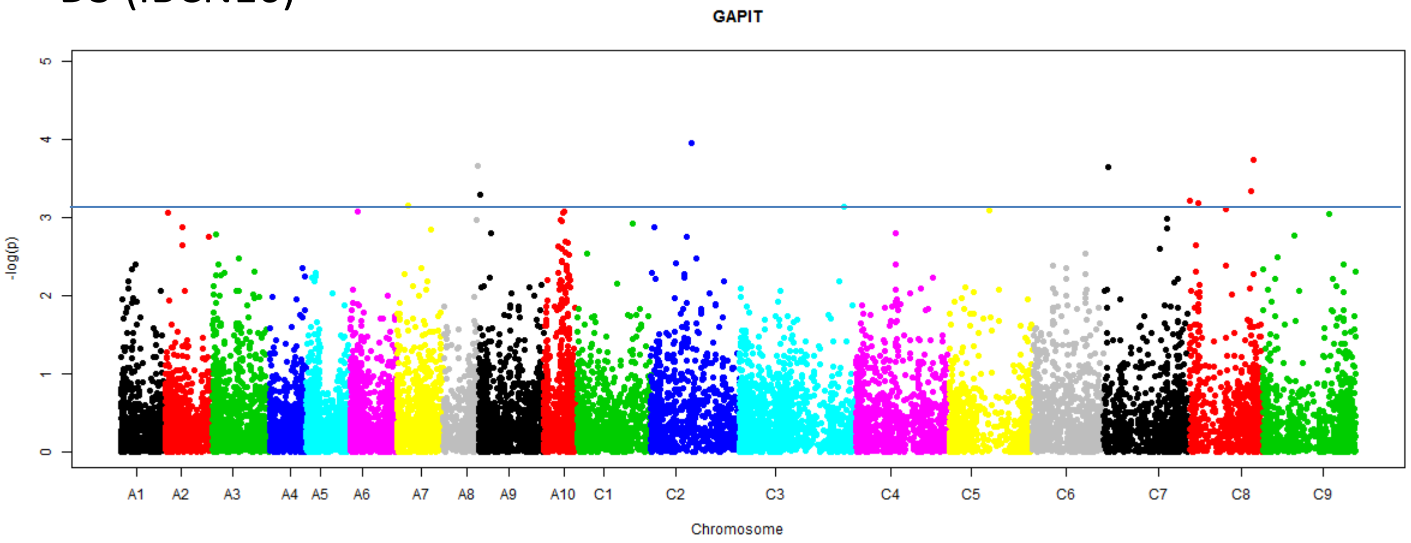

D4 (IBCN17)

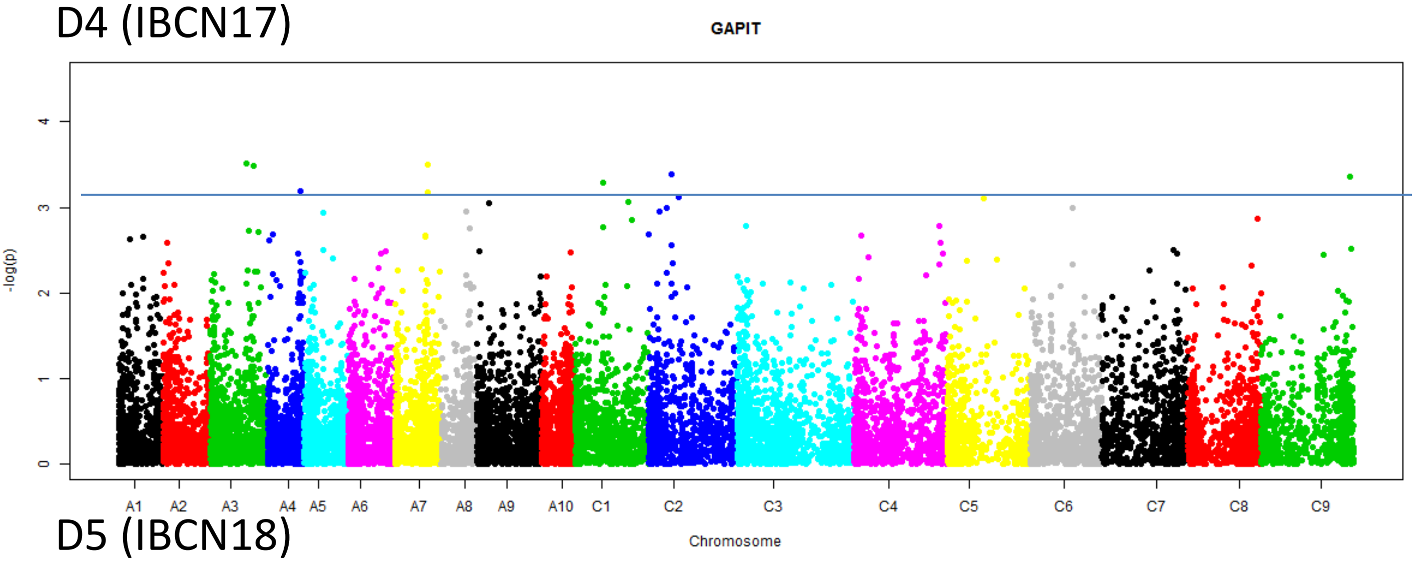

D5 (IBCN18)

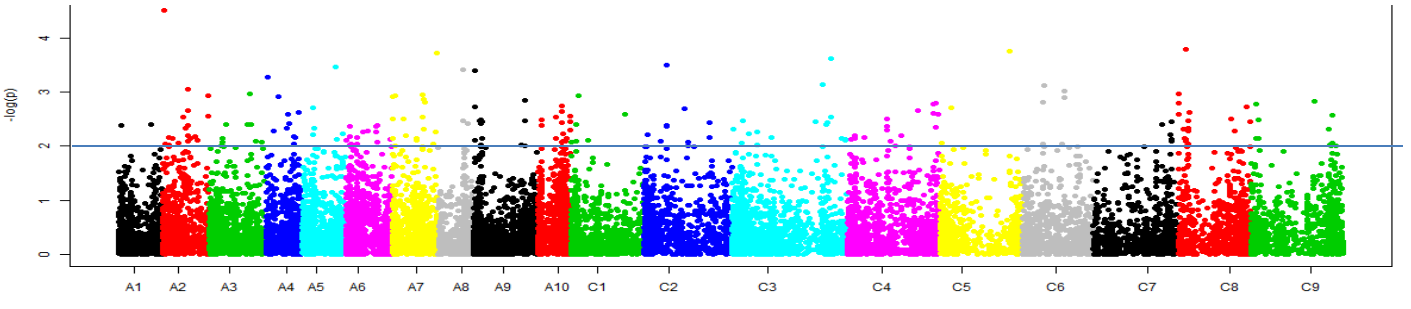

D6 (IBCN75)

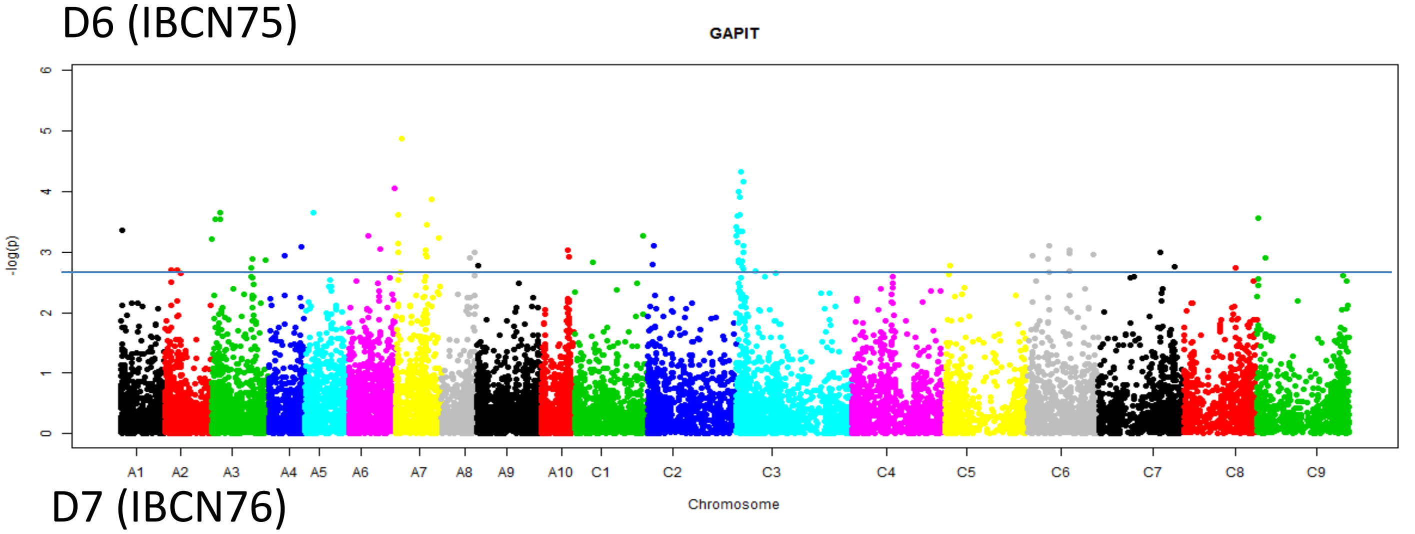

D7 (IBCN76)

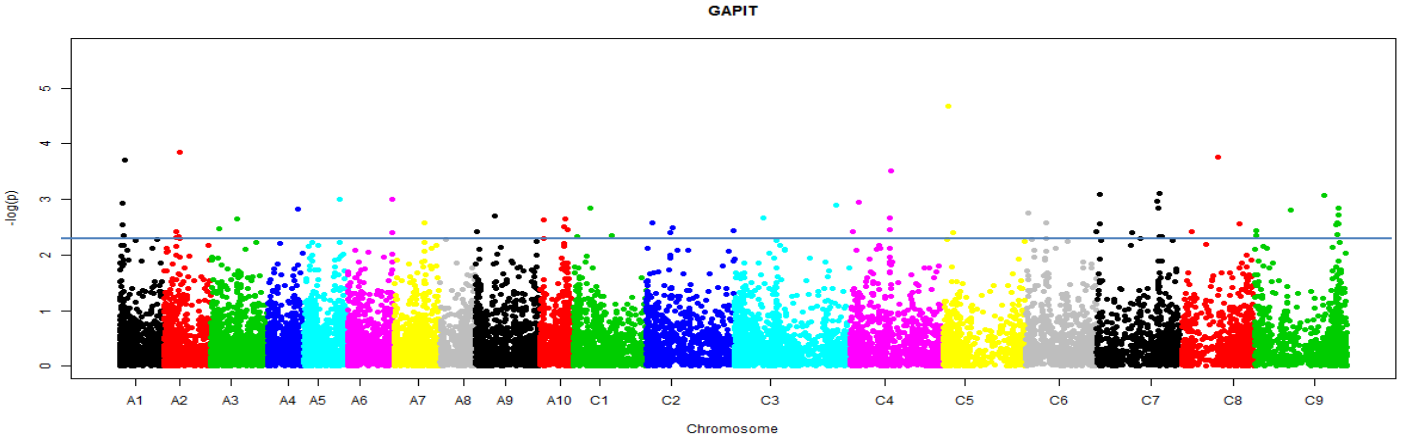

D8

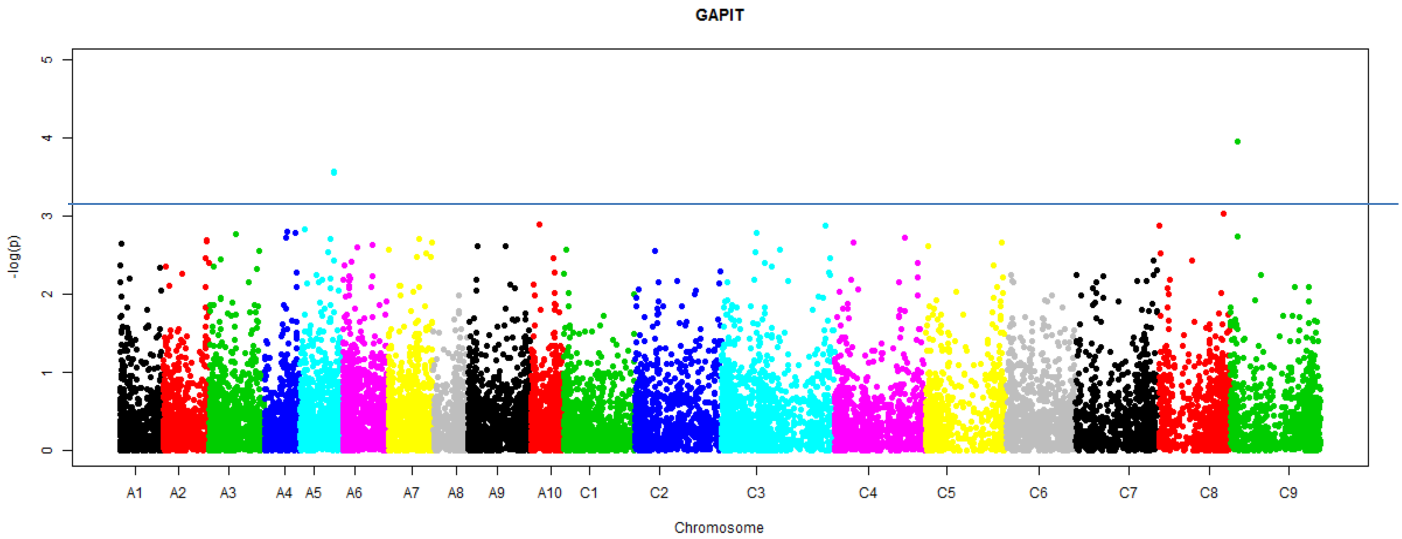

D9

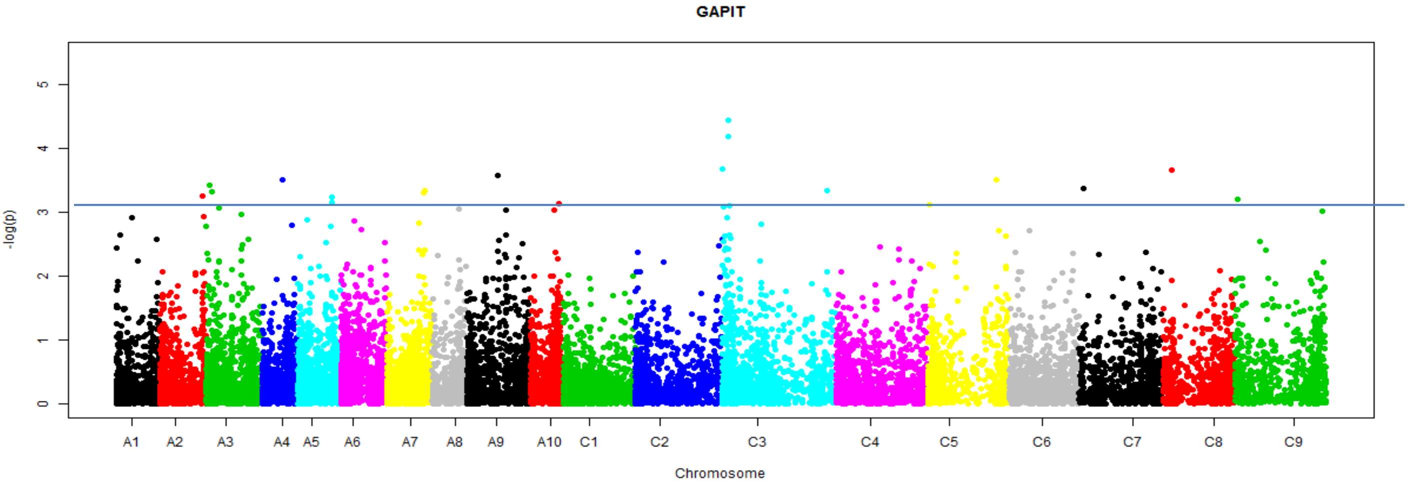

D10 (PHW1223)

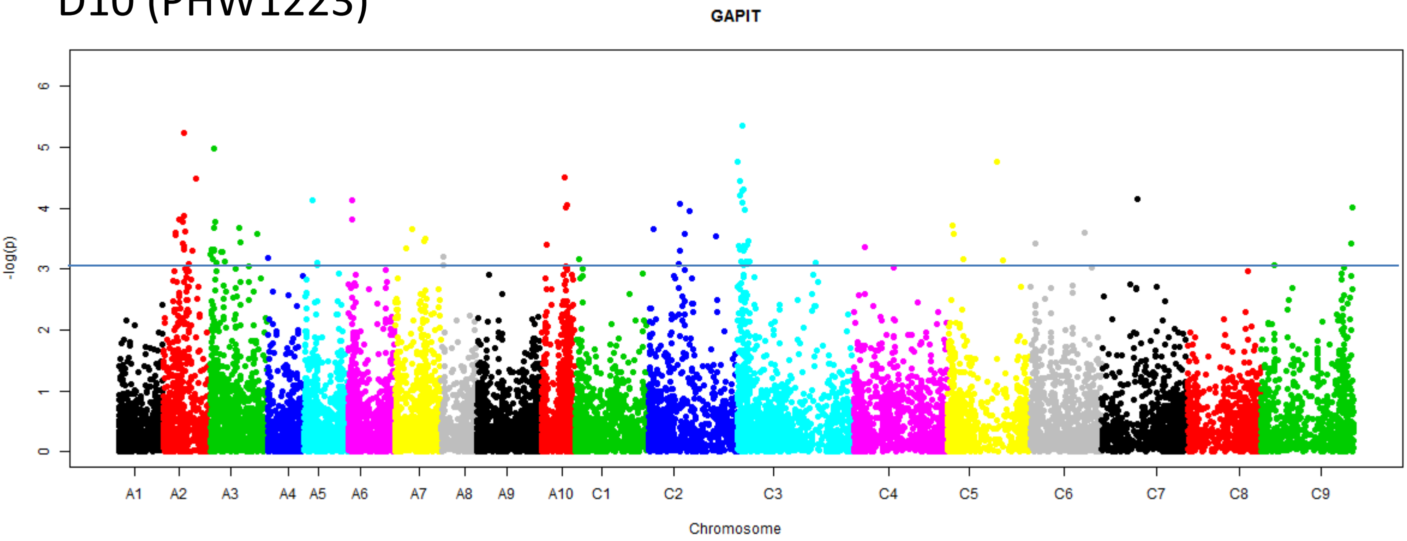

04MGPS021

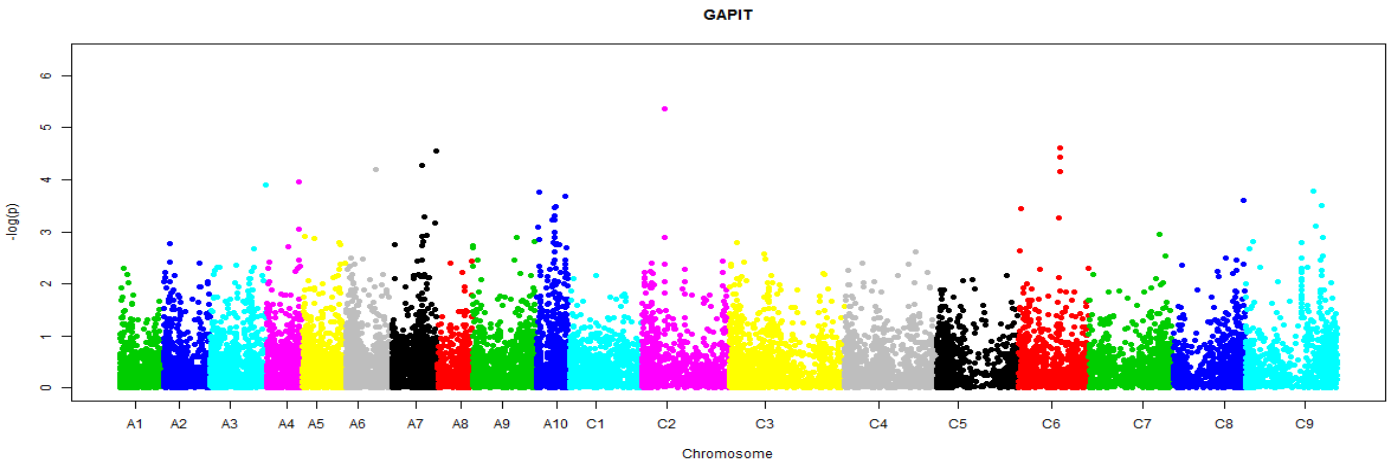

06MGPP041

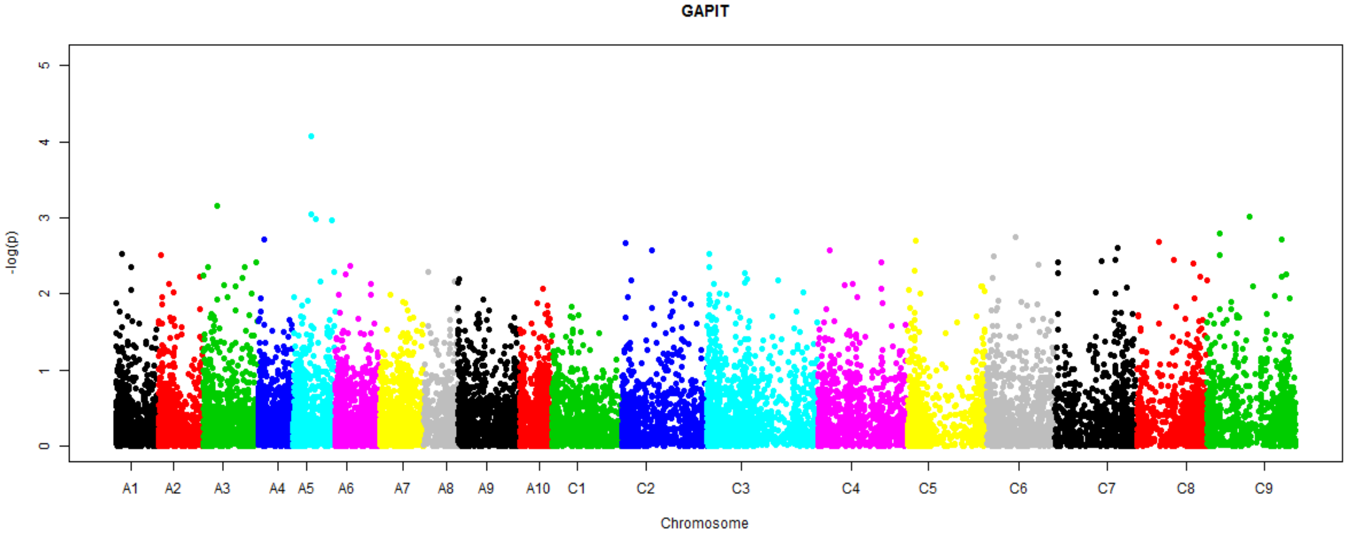

Supplement: Supplementary file 11 [file Image_2.pdf]
