# Supplementary material for: Genome-wide Association Study Identifies New Loci for Resistance to Leptosphaeria maculans in Canola
Source: Front Plant Sci. 2016 Oct 24;7:1513. doi: 10.3389/fpls.2016.01513 (PMC5075532; doi:10.3389/fpls.2016.01513)

Figure S3: Effect of subpopulation on the genome-wide associations for resistance to single spore isolate 04MGPS021 of *L. maculans.* Subpopulations were determined using principal component analysis as described in Raman H. et al. (2016). Manhattan plots show genome-wide *P* values for associations between SNP markers and resistance to *L. maculans* evaluated in full GWAS, subpopulations I and II. Different colours represent different chromosomes (A01-A10, C01-C9). Significant associations were tested at - log10(*p*) value of ≤ 3.

A - Full GWAS panel

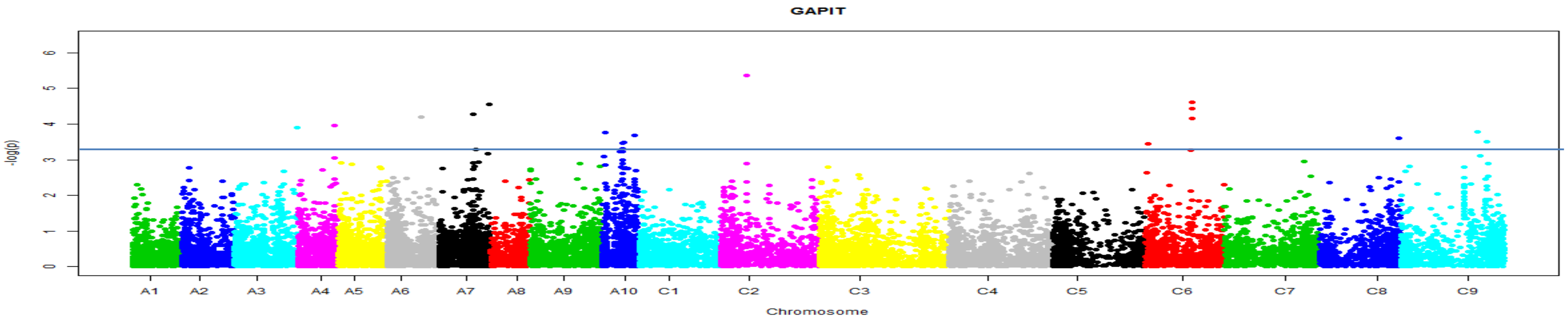

B - Subpopulation I

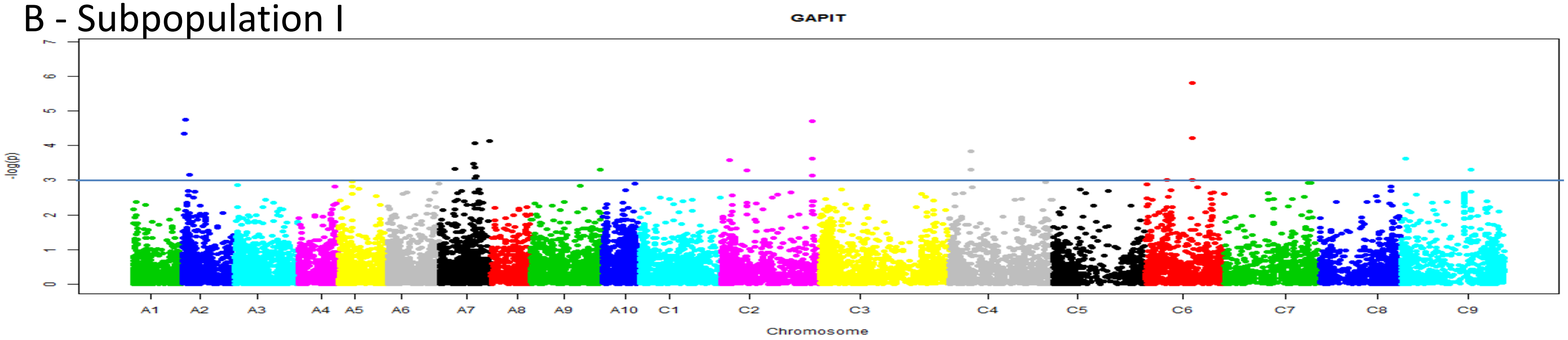

C - Subpopulation II

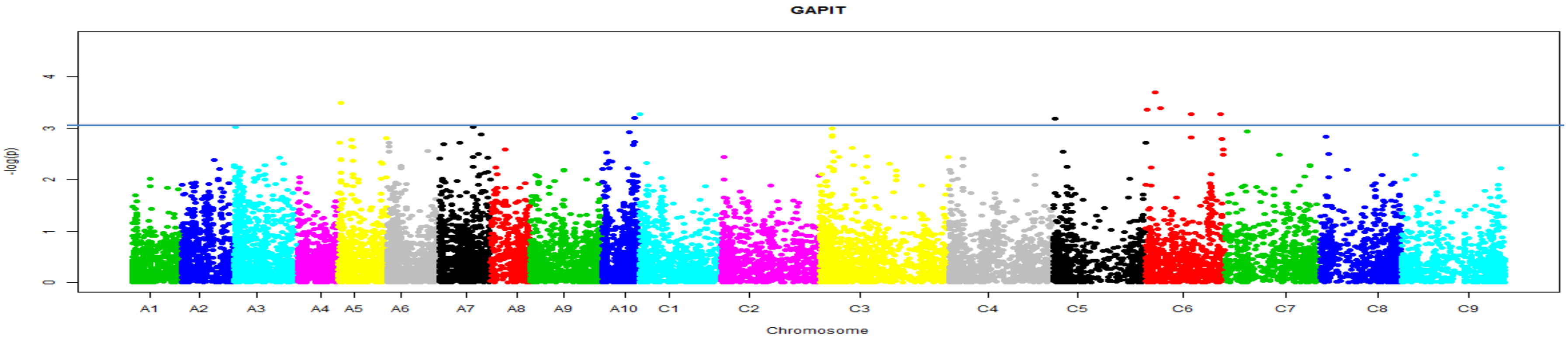

Supplement: Supplementary file 12 [file Image_3.pdf]
