# Supplementary material for: Genome-wide Association Study Identifies New Loci for Resistance to Leptosphaeria maculans in Canola
Source: Front Plant Sci. 2016 Oct 24;7:1513. doi: 10.3389/fpls.2016.01513 (PMC5075532; doi:10.3389/fpls.2016.01513)

Figure S4: Comparative genetic and physical mapping of molecular markers linked with *Rlm1* locus for resistance to *L. maculans* isolates D5 (IBCN18) and D7 (IBCN76) in a DH population from the Maxol*1/Westar-10. Resistance was assessed at the seedling stage (cotyledon) under glasshouse conditions at Wagga Wagga Agricultural Institute.

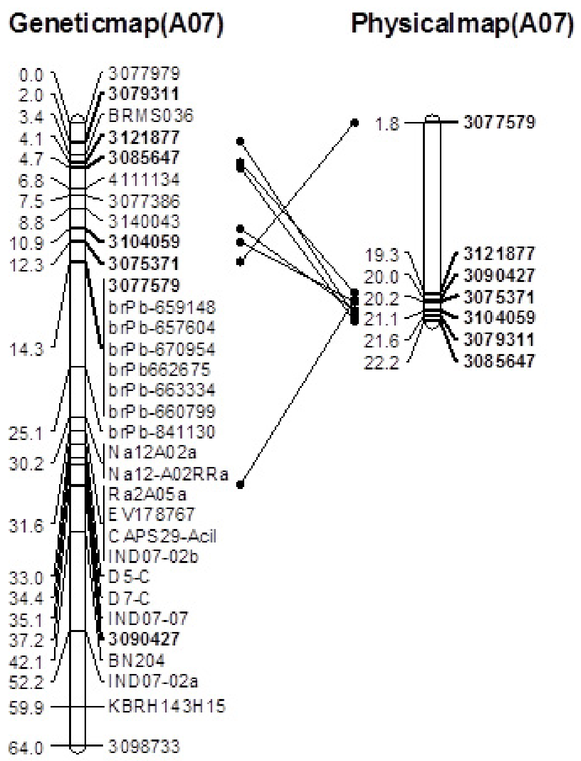

Supplement: Supplementary file 13 [file Image_4.pdf]
